# Differential benefits for temporal intervals and line segments with feedback and relevance to new learning with transfer effects

**DOI:** 10.64898/2026.07.26.740865

**Published:** 2026-07-29

**Authors:** Jiaxuan Teng, Arne D. Ekstrom, Eve A. Isham

## Abstract

Learning to accurately estimate temporal intervals and spatial magnitudes is important to everyday behavior, yet few studies have directly compared how learning unfolds across these domains. Theoretical models of temporal interval estimation provide conflicting accounts of how this learning process might occur and how it might differ from other entities like spatial line estimation. In the present study, participants completed temporal interval and line-length production tasks under feedback or no-feedback conditions during a training phase. They were then tested without feedback and subsequently presented with untrained temporal intervals and line lengths to examine whether learning generalized to novel magnitudes. We found that both temporal and spatial production exhibited similar learning trajectories, characterized by rapid initial improvements, followed by asymptotic performance. Despite similar learning trajectories, however, spatial production reached higher levels of accuracy and precision faster and in a more sustained manner than temporal production. Feedback facilitated learning in both domains, with improvements in precision emerging early during training and improvements in accuracy becoming evident during the subsequent test phase. These benefits persisted into the transfer phase, particularly for spatial production, indicating that feedback-supported learning generalized to untrained magnitudes. Transfer performance also reflected systematic biases in magnitude estimation, consistent with a central tendency effect, with shorter magnitudes tending to be overproduced and longer magnitudes underestimated. Together, these findings demonstrate that temporal and spatial magnitude learning share some common and some dissociable learning dynamics, providing new insight into the shared and distinct mechanisms underlying magnitude learning and highlighting the important role of feedback in promoting learning and transfer.

## Full Text Availability

The license terms selected by the author(s) for this preprint version do not permit archiving in PMC. The full text is available from the preprint server.

